# A Role for MOSPD1 in Mesenchymal Stem Cell Proliferation and Differentiation

**DOI:** 10.1002/stem.2102

**Published:** 2015-08-14

**Authors:** Madina Kara, Richard A. Axton, Melany Jackson, Sahar Ghaffari, Katrin Buerger, Alistair J. Watt, A. Helen Taylor, Brigid Orr, Winters R. Hardy, Bruno Peault, Lesley M. Forrester

**Affiliations:** ^1^ MRC Centre for Regenerative MedicineEdinburghUK; ^2^ Orthopaedic Hospital Research CenterUniversity of California at Los AngelesLos AngelesCaliforniaUSA

**Keywords:** MOSPD1, Embryonic stem cells, Mesenchymal stem cells, Proliferation, Differentiation

## Abstract

Mesenchymal stem cells (MSCs) isolated from many tissues including bone marrow and fat can be expanded in vitro and can differentiate into a range of different cell types such as bone, cartilage, and adipocytes. MSCs can also exhibit immunoregulatory properties when transplanted but, although a number of clinical trials using MSCs are in progress, the molecular mechanisms that control their production, proliferation, and differentiation are poorly understood. We identify MOSPD1 as a new player in this process. We generated MOSPD1‐null embryonic stem cells (ESCs) and demonstrate that they are deficient in their ability to differentiate into a number of cell lineages including osteoblasts, adipocytes, and hematopoietic progenitors. The self‐renewal capacity of MOSPD1‐null ESCs was normal and they exhibited no obvious defects in early germ layer specification nor in epithelial to mesenchymal transition (EMT), indicating that MOSPD1 functions after these key steps in the differentiation process. Mesenchymal stem cell (MSC)‐like cells expressing CD73, CD90, and CD105 were generated from MOSPD1‐null ESCs but their growth rate was significantly impaired implying that MOSPD1 plays a role in MSC proliferation. Phenotypic deficiencies exhibited by MOSPD1‐null ESCs were rescued by exogenous expression of MOSPD1, but not MOSPD3 indicating distinct functional properties of these closely related genes. Our in vitro studies were supported by RNA‐sequencing data that confirmed expression of *Mospd1* mRNA in cultured, proliferating perivascular pre‐MSCs isolated from human tissue. This study adds to the growing body of knowledge about the function of this largely uncharacterized protein family and introduces a new player in the control of MSC proliferation and differentiation. Stem Cells *2015;33:3077–3086*

**Significance Statement**

Mesenchymal stem cells are the subject of many clinical trials but the molecular mechanisms involved in their production, proliferation and differentiation are poorly understood. This study reveals that cells deficient in the MOSPD1 gene display distinct defects in mesenchymal stem cell proliferation and differentiation suggesting an important role for this poorly characterised gene family. Differentiation defects are rescued by expression of MOSPD1 but not MOSPD3 demonstrating that these two closely related genes are not functionally redundant.

## Introduction

Mesenchymal stem cells (MSCs) hold great promise for cell therapies because they can be easily isolated from accessible tissues such as bone marrow and fat and they can be expanded extensively in vitro. They can differentiate into bone, cartilage, and adipocytes and are proposed to hold immunotherapeutic properties upon transplantation 1. Their use in regenerative medicine has been widely proposed with a number of clinical trials in progress 2, 3. However, the molecular pathways that control their production, proliferation, and differentiation are not clearly defined.

Major sperm protein (MSP) domain‐containing proteins belong to a large family, which are found in yeast, plants and throughout the animal kingdom from insects to mammals 4, 5, 6, 7. They appear to have diverse roles in a variety of systems including nematode reproduction, synaptic transmission in the central nervous system in the sea slug and in the human neurodegenerative disease amyotrophic lateral sclerosis (ALS) 7, 8, 9. Included in this overarching MSP domain‐containing family are the four members of the MOSPD1 group (MOSPD1‐4), characterized by having an N‐terminal MSP domain and a number of predicted C‐terminal transmembrane domains. Functional studies have suggested that this subfamily is important during mammalian development. For example, phenotypic characterization of a mouse strain carrying a gene trap integration in the *Mospd3* locus suggested that *Mospd3* played a role in cardiac development with homozygous animals displaying a thinning of the right ventricular parietal wall 10, 11. More recently, the X‐linked *Mospd1* gene has been implicated in the process of epithelial to mesenchymal transition (EMT), an important event involved in many developmental and tumorogenic processes 12.

To provide insight into the function of Mospd1 in development and differentiation, we knocked out the *Mospd1* gene in mouse embryonic stem cells (ESCs) by gene targeting and tested the ability of these MOSPD1‐null cells to differentiate into a number of different cell lineages in vitro. We noted a deficiency in the differentiation of osteoblasts, adipocyte, and hematopoietic lineages and in the proliferative capacity of MSC‐like cells. The expression profile of *Mospd1* mRNA in MSCs and their precursors isolated from human tissue are consistent with our in vitro observations. MOSPD1‐null ESC differentiation deficiencies were rescued by exogenous expression of *Mospd1 cDNA*, but not *Mospd3*, indicating that these two closely related proteins have distinct functions.

## Materials and Methods

### Maintenance and Self‐Renewal of ESCs

All ESC lines were maintained in ESC media in the presence of serum and leukemia inhibitory factor (LIF) as described previously 13 and were subjected to regular karyotypic analysis. ESC self renewal assays were performed by plating 200 cells per milliliter in ESC medium for 5 days, then stained for alkaline phosphatase activity (Sigma‐Aldrich St Louis, MO, www.sigmaaldrich.com) to identify colonies classified as stem (pink), mixed (pink/white), or differentiated (white) as described previously 13.

### Production of Genetically Manipulated Cell Lines

The *Mospd1* targeting vector was generated by recombineering 14 using specific primers (Supporting Information Fig. S1, Table S1). The vector contained two lox P sites flanking exons 2‐4 of the *Mospd1* gene and an frt‐neomycin‐frt cassette in the intron between exon 4 and the 3′ lox P site. The full length *Mospd1* cDNA was cloned by reverse transcription polymerase chain reaction (RT‐PCR) from day 5 embryoid bodies (EBs) using specific primers (Supporting Information Table S1) and the *Mospd1* PCR product was cut with the restriction enzyme EcoR1 and cloned into the pCAG‐IRESpuro plasmid. Correctly orientated clones were sequenced and protein expression verified by Western blotting of transfected COS7 cell lysates. A pCMV6‐AN‐GFP vector containing the *Mospd3* gene was obtained from Origene, Rockville, MD (www.origene.com). Targeting and expression vectors were electroporated into E14 IV (referred to as E14) mouse ESCs using a Bio‐Rad gene pulser electroporator (0.5 cm cuvette at 0.25 kV and 500 μF capacitance), plated in ESC media plus LIF and selection was started the following day by adding 280 ng/ml G418 (concentration determined by kill curve) for 10 days. Single G418‐resistant ESC colonies were picked, expanded, and DNA isolated from each colony was screened by Southern blot analysis. COS7 cells were transfected using Lipofectamine 2000 (Invitrogen (Waltham, MA, www.lifetechnologies.com)) following manufacturer's instructions.

### Differentiation Assays

Differentiation assays are outlined in Supporting Information Figure S2. All EB‐based assays followed a similar protocol in the initial stages where ESCs were cultured in hanging drops (300 cells per 10 μl drop) in LIF for 2 days and resulting EBs were harvested and cultured in suspension, in the absence of LIF, for varying numbers of days. For cardiomyocyte differentiation, EBs were cultured in suspension for 6 days, individual EBs were plated in gelatinized wells and the presence of beating regions was scored over the following 10 days. Hematopoietic differentiation was carried out by culturing EBs in suspension for 1 day in the absence of LIF (day 1 EBs) before plating onto gelatinized plates for a further 6 days. Cells were then dissociated and plated in methylcellulose containing stem cell factor (SCF), erythropoietin (EPO), and interleukin 3 (IL3) as described previously 13 and colonies counted after a further 10–15 days.

For the colony forming unit‐fibrobast (CFU‐F) assay, day 1 EBs were plated onto gelatinized dishes and cultured for a further 4 days before being dissociated and plated in Mesencult medium (STEMCELL Technologies, Cambridge UK (www.stemcell.com)) for 14 days then stained with Leishman stain. Mesenchymal cells from the CFU‐F assays were grown for up to nine passages in Mesencult medium and their phenotype confirmed by flow cytometry with antibodies against CD90, CD73, and CD105.

Osteoblast differentiation was performed as described 15 with day 1 EBs plated onto gelatinized plates for 4 days then dissociated to single cells and 5 × 10^4^ cells were cultured in 5 ml Alpha Minimal Essential Media (MEM) (Invitrogen) supplemented with 15% fetal calf serum (FCS), 0.05 mM β‐mercaptoethanol, and 2 mM glutamine on gelatinized six‐well dishes for a further 14 days. At this time point, the medium was supplemented with 50 mM β‐glycerophosphate, 50 µg/ml ascorbic acid, and 1 µM dexamethasone and cultured for a further 7 days. Cells were washed, fixed in 70% ethanol then stained with 10 mg/ml Alizarin Red (Sigma‐Aldrich) 12 days later (day 26).

Adipocyte differentiation was carried out by treating day 1 EBs with 10^−5^ M retinoic acid (RA) from days 2–5 with medium and RA being replenished at day 4. EBs were plated onto gelatin at day 7 and treated with 5 µg/ml, 500 nM 3‐isobutyl‐1‐methylxanthine (IBMX) and 400 ng/ml dexamethasone and 5 μg/ml insulin. Medium was replenished every 2 days until day 20 when they were stained with oil red O. For chondrocyte differentiation, day 5 EBs were plated in medium containing 15% FCS which was replaced every 2 days until being stained with Alcian Blue after 19 days.

### Real‐Time Quantitative RT‐PCR

Total RNA was isolated using the RNeasy Mini kit (Qiagen, Manchester, UK, www.qiagen.com) and 500 ng of total RNA was reverse‐transcribed using the Superscript III kit (Invitrogen). cDNA was subjected to quantitative RT‐PCR (qRT‐PCR) using specific primers and either Roche universal library or TaqMan probes which were all prevalidated before use (Supporting Information Table 1). The ABI 7500 FAST qPCR machine and SDS v1.4 software (Applied Biosystems, Waltham, MA [www.appliedbiosystems.com]) were used with relative quantification being calculated using the delta delta CT method and *Hprt* as an endogenous housekeeping gene.

### Generation of Monoclonal Antibodies

A monoclonal α‐MOSPD1 antibody was generated against the chemically synthesized peptide MHQQKRQPELVEGNLPVFVF (Thistle Research, Glasgow, Scotland (www.peptides.co.uk) and Yorkshire Biosciences, York, UK (http://www.york-bio.com)). The resulting hybridoma cells were cultured in RPMI‐1640 medium (Invitrogen) supplemented with 10% heat inactivated FCS, 2 mM l‐glutamine, and 100 units penicillin–100 µg/ml streptomycin for 2 weeks at 37°C with 5% CO_2_ without changing the medium. This medium, containing α‐MOSPD1 antibody, was used for subsequent immunological experiments.

### Preparation and Analysis of Protein Lysates

Protein samples were prepared and analyzed by SDS PAGE and Western blotting according to standard procedures. Briefly, cells were washed with phosphate‐buffered saline (PBS), lysed in ice‐cold RIPA buffer (ThermoScientific, Waltham, MA, www.thermoscientific.com), scraped from the plate, passed through a 19G needle, washed in PBS then sonicated in the presence of protease inhibitors (Sigma‐Aldrich). Lysates were run on a 12% SDS/polyacrylamide electrophoresis gel (Bio‐Rad, Hercules, CA, www.bio-rad.com) then transferred to nitrocellulose membranes by semi‐dry electrotransfer. Membranes were blocked for 1 hour with 5% dried milk powder in 1× TBST (25 mM Tris‐HCl, pH 8.0, 125 mM sodium chloride, 0.1 % Tween 20) before hybridization with the primary antibodies (1:100 α‐MOSPD1 and α‐MOSPD3, 1:2,000 α‐GAPDH [Abcam, Cambridge, UK, www.abcam.com]) in blocking solution overnight at 4°C. The membranes were washed in TBST and incubated with horseradish peroxidase‐conjugated secondary antibody (1:2,000) for 1 hour at room temperature before adding ECL substrate and autoradiography.

### Isolation of Human MSC Progenitors from Fat Tissue

Lipoaspiration specimens used for FACS sorting, culture, and RNA‐SEQ analysis were obtained from six healthy female donors (age range: 35–63 years; BMI range: 21.1–32.0) undergoing elective liposuction of fat from lateral axillary areas at local plastic surgery clinics. Lipoaspirates stored overnight at 4°C were washed twice with PBS lacking Ca and Mg to reduce contamination by blood and then combined with an equal volume of Dulbecco's modified Eagle's medium (DMEM) medium (Lonza, Basel, Switzerland, www.lonza.com ) containing antibiotics, 3.5% (w/v) bovine serum albumin (Hycult Biotech, www.hycultbiotech.com), and 1 mg/ml collagenase (cat. #C‐6685, Sigma‐Aldrich). Digestion proceeded for 40 minutes at 37°C during vigorous shaking (250 rpm) then the stromal vascular fraction was processed as described previously 16. Following cell counting using a hemocytometer and trypan blue to assess viability, the SVF was stained for 40 minutes at 37°C in DMEM medium lacking fetal bovine serum (FBS) with 2 µg/ml Hoechst 34580 (Life Technologies, Waltham, MA, www.lifetechnologies.com), and Aldefluor (StemCell Technologies) according to the manufacturer's instructions, to compare cytoplasmic levels of aldehyde dehydrogenase (ALDH) in nucleated cells. The Aldefluor‐stained cells, and a control containing an ALDH inhibitor (ALDH‐neg control), were centrifuged at 300*g* for 5 minutes and resuspended in 1 ml of Aldefluor assay buffer containing 2 µl of Near infrared live/dead stain (Life Technologies) and 10 µl of FcR blocking reagent (Miltenyi Biotec, Bergisch Gladbach, Germany, www.miltenyibiotec.com), and incubated for 10 minutes on ice. The cells were collected by centrifugation and incubated in Aldefluor assay buffer containing fluorescently labeled, anti‐human, mouse monoclonal antibodies (PE‐ or APC‐Cy7‐conjugated CD45, APC‐CD34, and PE‐ or PerCP‐Cy5.5‐conjugated CD146; BD BioSciences, San Jose, CA, www.bdbiosciences.com) for 20 minutes on ice to allow immunophenotypic staining for fluoresecence activated cell sorting (FACS) (Supporting Information Fig. S4). Cells collected by FACS were either lysed immediately for RNA extraction or cultured for three passages in DMEM containing antibiotics and 20% FBS. To adjust for gene expression changes brought about by cell sorting, FACS‐selected cultured adventitial vascular cells (ASC) and pericytes were stained with fluorescent antibodies to CD31, CD45, CD146, and CD34 after three passages. All cultured ASCs were collected as they demonstrated no expression for any of the above markers; however, only pericytes that had retained CD146 expression in culture were isolated for RNA‐SEQ analysis.

### RNA Purification and RNA Sequencing

Subpopulations consisting of freshly isolated total ASC, ALDH‐dim and ‐bright ASC, and ALDH‐dim pericytes and their cultured counterparts were sorted into cell lysis solution and purified using the Qiagen RNeasy Micro kit (Qiagen). Total RNA was quantified and yielded RNA integrity numbers of 7.7 or greater (2100 Agilent Bioanalyzer, www.genomics.agilent.com). cDNA for each subpopulation was prepared using the NuGen's Ovation RNA‐Seq System V2 kit from approximately 100 ng of RNA pooled from 2 or 3 donors and used as input for Ovation's Ultralow DR Multiplex System 1–8 (Nugen, San Carlos, CA, www.nugen.com). High throughput sequencing was conducted on an Illumina Hiseq 2000 platform to generate 100‐bp paired‐end reads at a depth of coverage of approximately 79 million reads. Cluster formation achieved approximately 86%, while the quality of reads exceeded 95%. Fragment sequence identification and alignment were performed using TopHat and Bowtie, respectively. Samples were normalized by RPMK (reads per kilobase of exon per million mapped reads) which takes into account both library size and gene length in within‐sample comparisons. Similar numbers of reads between libraries (i.e., depths of coverage) made cross‐library, individual gene comparisons possible.

### Statistical Analysis

Quantifiable data were graphed and analyzed with the appropriate statistical tests (*t*‐test or analysis of variance) using GraphPad Prism (GraphPad Software, San Diego, CA, http://www.graphpad.com). Analysis was performed on triplicate data sets unless otherwise stated.

## Results

### Generation of MOSPD1‐Null ESCs

**Figure 1:**
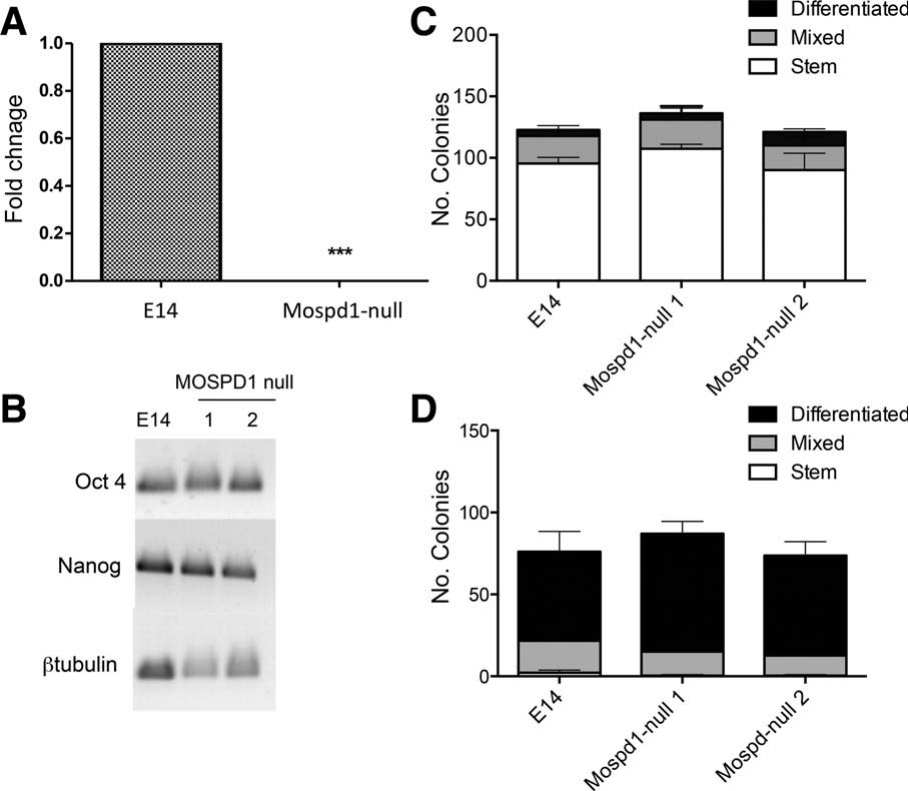
Self‐renewal and spontaneous differentiation of MOSPD1‐null embryonic stem cells (ESCs) are comparable with control E14 ESCs. **(A):** Quantitative reverse transcription polymerase chain reaction (qRT‐PCR) analysis confirmed the lack of expression of *Mospd1* transcripts in differentiating day 5 embryoid bodies generated from two independently derived MOSPD1‐null ESC clones compared with control E14 (Wild type) ESCs. Data shown is the mean for the two cell lines (*p* < 0.001). **(B):** Semi‐quantitative RT‐PCR demonstrated that the stem cell markers *Oct4* and *Nanog* were expressed in two independently‐derived MOSPD1‐null ESCs (1 and 2) at a comparable level to control E14 ESCs. β‐*Tubulin* was used as a loading control. **(C):** The number of stem, mixed or differentiated colonies generated from MOSPD1‐null ESCs in an alkaline phosphatase clonal density assay in the presence of leukemia inhibitory factor (LIF) was not significantly different (*p* = 0.4) to control E14 ESCs. (Data represent the means of two independently derived MOSPD1‐null cell ESC lines). **(D):** The number of stem, mixed, or differentiated colonies generated from MOSPD1‐null ESCs in an alkaline phosphatase clonal density assay in the absence of LIF was not significantly different (*p* = 0.26) to control E14 ESCs. (Data represent the means of two independently‐derived MOSPD1‐null cell ESC lines).

A conditional *Mospd1* targeting vector was designed with loxP sites flanking exons 2–4 of the *Mospd1* gene, an frt‐neomycin‐frt cassette inserted into the intron between exon 4 and the 3′ loxP site with appropriate restriction enzyme sites to differentiate between wild type, targeted floxed, and the subsequently excised null alleles (Supporting Information Fig. S1A). The targeting vector was electroporated into E14IV ESCs then G418‐selected colonies were isolated and analyzed to identify ESCs carrying the correctly targeted *Mospd1*‐floxed allele (*Mospd1* ^*tm1floxFor*^) (Supporting Information Fig. S1B). Briefly, genomic DNA was isolated from 179 G418‐resistant ESC clones and Spe1 and Hind111 digested DNA was assessed by Southern blotting using probes that were 5′ and 3′ of the targeting vector. We identified four ESC clones that contained the restriction fragments diagnostic of correct targeting implying a 2% targeting efficiency (Supporting Information Fig. S1B). The pCAGGS‐Cre‐IRESpuro plasmid 17 was electroporated into a number of correctly targeted clones to excise exons 2–4 and the frt‐neo‐frt cassette to produce ESCs carrying the *Mospd1*‐null allele (*Mospd1* ^*tm1fFor*^) (Supporting Information Fig. S1C). As *Mospd1* is X‐linked, targeting of the one allele in male E14 ESCs generates a functionally null ESC line with no *Mospd1* mRNA expression. This was confirmed by quantitative RT‐PCR analysis of RNA isolated from differentiating wild type and MOSPD1‐null ESCs (Fig. 1A). All of the following functional studies were performed on two MOSPD1‐null ESC clones that were derived from independently derived targeted ESC lines.

### MOSPD1 Deficiency Has No Effect on the Maintenance of Undifferentiated ESC

MOSPD1‐null ESCs could be maintained as undifferentiated cells in the presence of LIF in a comparable manner to control E14 ESCs and expressed comparable levels of the stem cell markers, *Oct4* and *Nanog* (Fig. 1B), indicating that *Mospd1* was not essential for ESC self‐renewal. We confirmed this in a quantitative manner using an alkaline phosphatase clonal density assay (Fig. 1C). No significant difference was observed in the number of stem cell colonies formed in the presence of LIF between control E14 and MOSPD1‐null ESCs indicating that self‐renewal was not dependent on MOSPD1. A comparable number of differentiated and mixed colonies were also observed indicating that MOSPD1‐null cells displayed a similar degree of spontaneous differentiation in the presence of LIF (Fig. 1C). When this clonal assay was performed in the absence of LIF there was no significant difference in the morphology, nor the numbers of differentiated colonies produced between control E14 and MOSPD1‐null ESCs indicating that the early ESC differentiation events were also *Mospd1* independent (Fig. 1D). Two independently‐derived MOSPD1‐null ESC clones (MOSPD1 null 1 and MOSPD1 null 2) behaved in a similar manner and the data shown represent the mean of the two clones.

**Figure 2:**
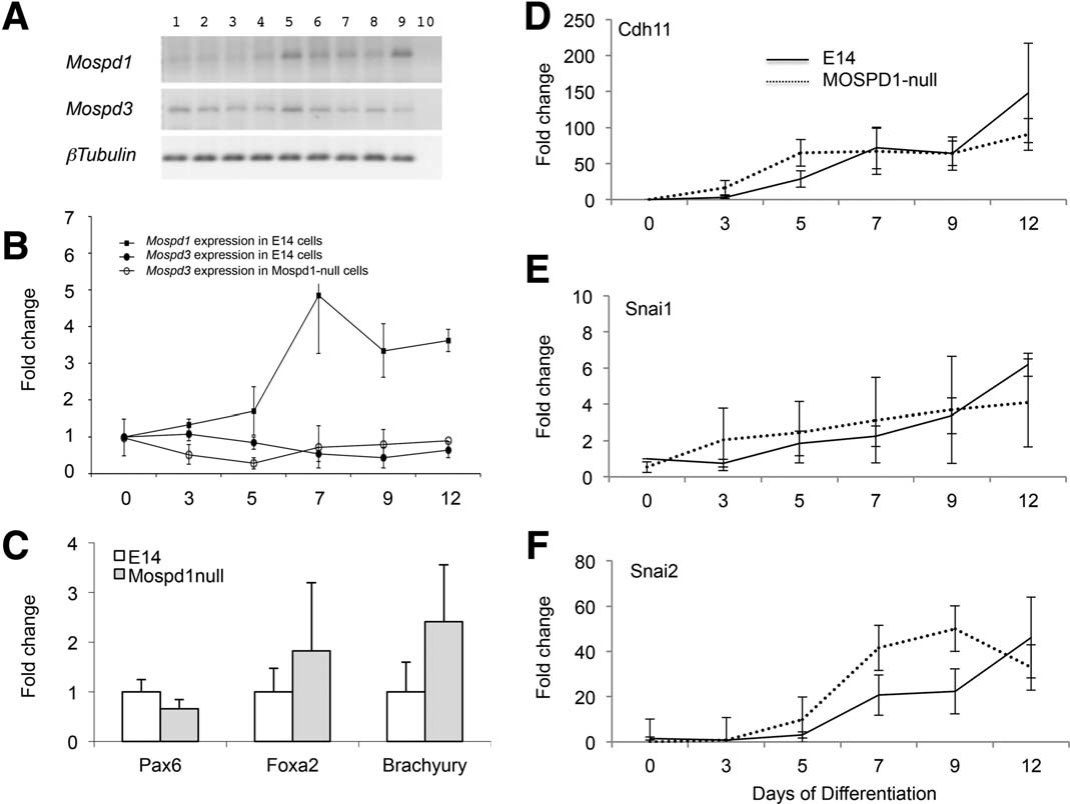
*Mospd1* expression increases during embryonic stem cell (ESC) differentiation but is not required for germline specification nor epithelial to mesenchymal transition (EMT). **(A):** Semi‐quantitative reverse transcription polymerase chain reaction (RT‐PCR) analysis of RNA isolated from undifferentiated control E14 ESCs (lanes 1 and 2) and in ESCs differentiated in the absence of leukemia inhibitory factor (LIF) for 1, 2, 3, 4, and 5 days (lanes 3–7, respectively) and in day 12 embryoid bodies (EBs) (lanes 8 and 9) using primers complementary to *Mospd1, Mospd3*, and *β‐tubulin* as a loading control. Lane 10 represents a negative control with no RNA loaded. **(B):** Quantitative RT‐PCR analysis of RNA isolated from control E14 and MOSPD1‐null undifferentiated ESCs (day 0) and from cells that had differentiated in the absence of LIF for 3, 5, 7, 9, and 12 days using primers specific for *Mospd1* and *Mospd3*. Data are expressed as fold expression relative to the day 0 time point of each assay. **(C):** Quantitative RT‐PCR analysis of day 3 EBs isolated from control E14 and MOSPD1‐null ESCs using primers for germ lineage‐specific markers, *Pax6* (ectoderm), *Foxa2* (endoderm), and *Bry* (mesoderm*)*. **(D–F):** The expression of markers associated with EMT, *Cdh11* **(D)**, *Snai1* **(E)**, and *Snai2* **(F)** increase during the time course of differentiation to a comparable extent in control E14 and MOSPD1‐null ESCs.

We analyzed the expression of *Mospd1* mRNA and its close relative *Mospd3* in undifferentiated ESCs and in ESCs during EB differentiation by RT‐PCR (Fig. 2A, 2B). *Mospd1* is expressed at a relatively low level in undifferentiated ESCs (virtually undetectable by RT‐PCR) and its level of expression increases significantly during differentiation. The closest homologue of *Mospd1* is *Mospd3* and expression of this gene was detected at a higher level in ESCs and remains at a consistent level throughout the differentiation protocol (Fig. 2A). qRT‐PCR analysis confirmed these findings with a significant increase in *Mospd1* expression upon differentiation but a consistent level of *Mospd3* throughout the time period (Fig. 2B). Given the sequence homology between *Mospd1* and *Mospd3*, we compared the expression of *Mospd3* in *Mospd1*‐null ESCs compared with wild type ESCs throughout the differentiation process. Although there was a slightly lower level of expression of *Mospd3* in *Mospd1*‐null ESCs compared with control ESCs at the early stages of differentiation (days 3 and 5) these differences were not significant and at later stages these minor differences were resolved (Fig. 2B). Thus we can conclude that the loss of *Mospd1* expression is not compensated by an upregulation of its close homologue *Mospd3* and that expression of *Mospd3* is not absolutely dependent on *Mospd1* (Fig. 2B).

### MOSPD1‐Null ESCs Can Differentiate into all Three Germ Layers

To further characterize the effects of MOSPD1 deficiency, we differentiated control E14 and MOSPD1‐null ESCs in EBs in the absence of LIF for 3 days 13 and assessed the expression of genes that were upregulated during the formation of the three primary germ layers but were not expressed in undifferentiated ESCs (Fig. 2C). There was no significant difference in the expression of mesoderm (*T/Bry*), endoderm (*Foxa2*), or ectoderm (*Pax6*) markers indicating that all three germ layers could be formed in the absence of *Mospd1*. A previous study had suggested that *Mospd1* played a role in EMT 12 so we compared the expression of a number of genes associated with EMT during the differentiation process (Fig. 2D–2F). *Snai1, Snai2*, and *Cdh11* were all upregulated to a comparable extent during the differentiation of control E14 and MOSPD1‐null ESCs indicating that MOSPD1 does not play a major role in EMT in this system.

### MOSPD1‐Deficiency Impairs Differentiation of Some Mesodermal Cell Lineages

We next analyzed the ability of MOSPD1‐null cells to differentiate into a number of mesodermal lineages using a range of assays (summarized in Supporting Information Figs. S2, S3). Differentiation protocols were all initiated by the formation of EBs with the subsequent steps varying depending on the specific cell lineage to be analyzed.

**Figure 3:**
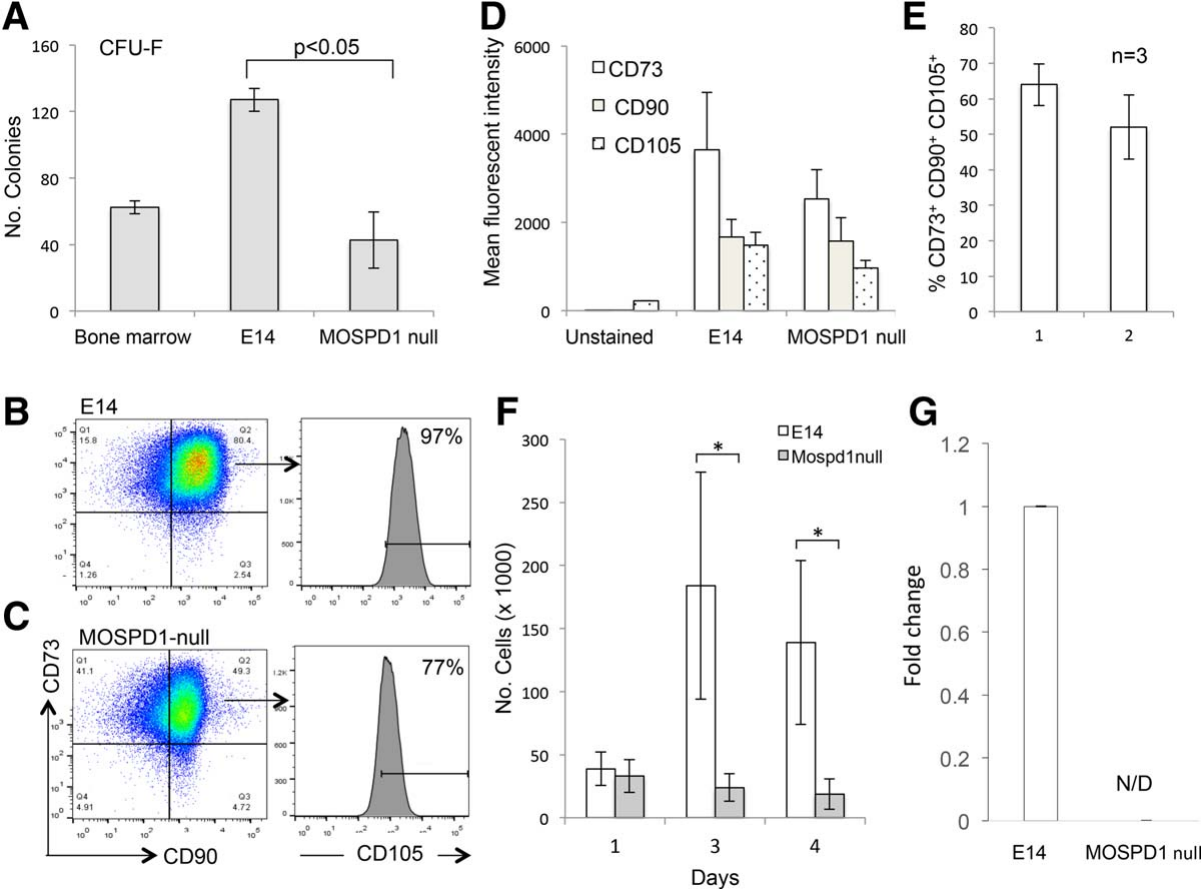
MOSPD1‐null cells are deficient in production and proliferation of MSC‐like cells. **(A):** The number of colony forming unit‐fibrobast (CFU‐F) colonies generated from MOSPD1‐null embryonic stem cell (ESC) lines is significantly lower than the number generated from control E14 ESCs. A cell suspension isolated from mouse bone marrow was used as a positive control for the CFU‐C assay. **(B, C):** Flow cytometry analyses of control E14 **(B)** and MOSPD1‐null **(C)** cells after nine passages in Mesencult media using antibodies against CD90 and CD73. The proportion of cells expressing CD105 was lower on CD90^+^/CD73^+^ cells derived from MOSPD1‐ null cells compared with control E14 cells. **(D,E):** The mean fluorescent intensity of CD90, CD73, and CD105 staining **(D)** and the proportion of cells expressing these markers **(E)** was lower in higher in MOSPD1‐null‐derived compared with control E14‐derived MSC‐like cells. **(F):** CD90^+^CD73^+^CD105^+^ MSC‐like cells derived from MOSPD1‐null ESCS proliferate at a slower rate compared with control E14 ESCs. Cells were grown in mesencult media for a number of passages, then plated in this assay at 50,000 per square centimeter then counted 1, 3, and 4 days later. (*, *p* = 0.03) **(G):** Quantitative reverse transcription polymerase chain reaction analysis of MSC‐like cells derived from control and MOSPD1‐null ESC‐derived MSC cells confirming complete absence of *Mospd1* transcripts. Abbreviation: CFU‐F, colony forming unit‐fibrobast.

Given previous reports on the role of MOSPD1 in the differentiation of mesenchymal lineages 12, we tested the ability of MOSPD1‐null cells to form colonies in medium designed to identify mesenchymal progenitors from bone marrow, known as CFU‐F 18. As expected, this assay identified a number of colonies from bone marrow (Fig. 3A). We compared control E14 and MOSPD1‐null ESCs in this assay and observed a significant reduction in the production of colonies generated from MOSPD1‐null ESCs (Fig. 3A). To address whether this assay does actually measure MSCs derived from ESCs we further cultured cells derived from these colonies in Mesencult medium. A population of cells expressing CD90, CD73, and CD105 emerged from both control and MOSPD1‐null cells but cells derived from MOSPD1‐null ESCs expressed slightly lower levels of these markers than control E14 cells (Fig. 3B–3E). We also noted that the cells that arose from the MOSPD1‐null cells took a longer time to reach confluency indicative of a reduced proliferation rate. This finding was confirmed in a quantitative manner by demonstrating that MOSPD1‐null cells failed to proliferate when plated at a defined density (Fig. 3F). The lack of expression of *Mospd1* mRNA transcripts was confirmed in MOSPD1*‐*null MSC‐like cells by qRT‐PCR (Fig. 3G).

**Figure 4:**
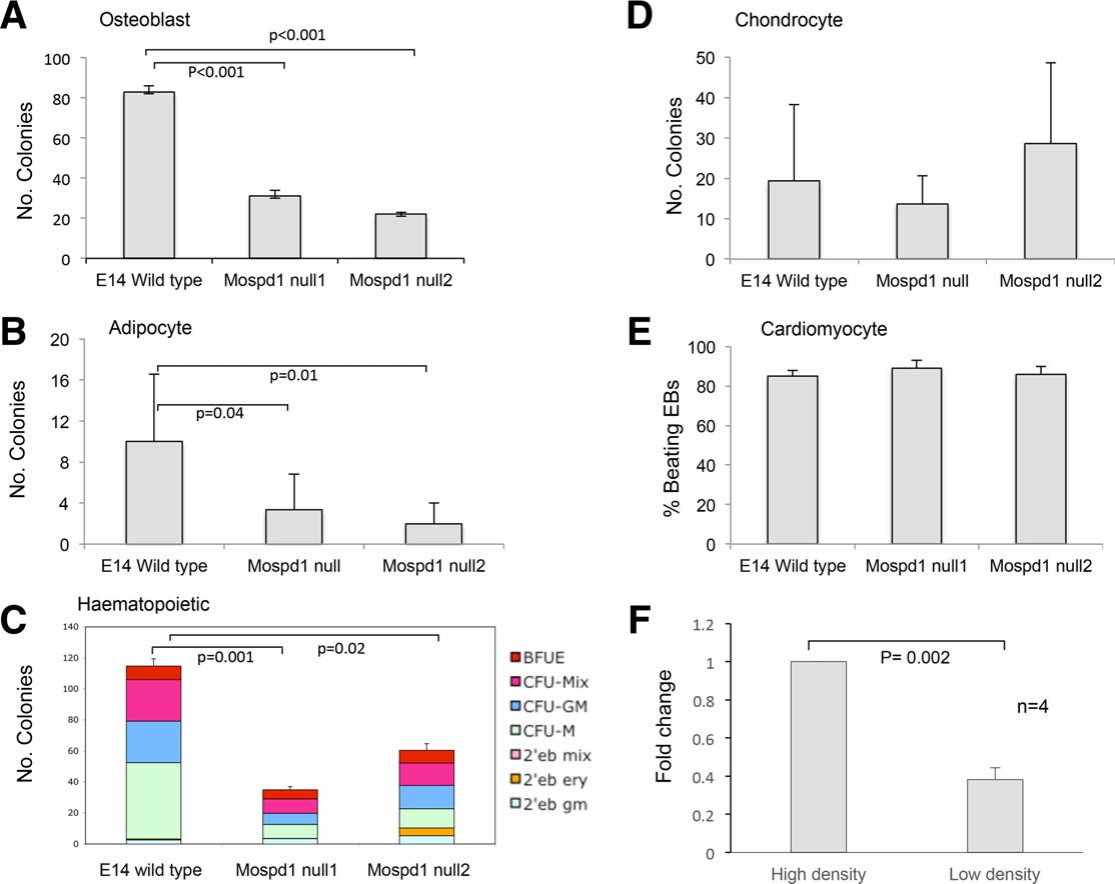
*MOSPD1‐null* embryonic stem cells (ESCs) exhibit differentiation defects. The differentiation potential of control E14 ESCs and MOSPD1‐null ESCs was assessed in a range of assays (Supporting Information Figs. S2, S3). There was a significant deficiency in the production of osteoblasts **(A)**, adipocytes **(B)**, and hematopoietic progenitors **(C)** in two independently‐derived MOSPD1‐null ESCs (MOSPD1‐null 1 and MOSPD1‐null 2) compared with control E14 ESCs. No significant difference was observed in the production of chondrocytes **(D)** nor beating cardiomyocytes **(E)** between control and MOSPD1‐null cells. **(F):** Quantitative reverse transcription polymerase chain reaction analysis of Mospd1 expression in MSC‐like cells derived from control E14 ESCs grown at high and low density. Cells were plated at 2,500 cells per square centimeter (low density) or 50,000 cells per square centimeter (high density) for 24 hours before harvesting and isolation of RNA.

Our hypothesis that MOSPD1‐null ESCs are deficient in their production of mesenchymal lineage cells is supported by the observation that MOSPD1‐null ESCs are also deficient in the production of osteoblasts (Fig. 4A). The ability of MOSPD1‐null ES cells to differentiate into cells that stained positive with the histochemical stain Alizarin Red that marks bone mineralization 15 was significantly reduced compared with control E14 ESCs (Fig. 4A; Supporting Information Fig. S3). Adipocyte differentiation was also reduced in MOSPD1‐null cells as assessed by culturing EBs in the presence of RA, IBMX, and insulin and staining with oil red O (Fig. 4B; Supporting Information Fig. S3). We also observed a significant reduction in the production of hematopoietic progenitors from MOSPD1‐null ESCs compared with control cells (Fig. 4C).

Given that MSCs give rise to osteoblasts, adipocytes, and chondrocytes, it is interesting that we did not observe any significant reduction in the production of chondrocytes derived from MOSPD1‐null ESCs compared with control E14 ESCs (Fig. 4D; Supporting Information Fig. S3). However, it is noteworthy that the chondrocyte differentiation protocol is relatively non‐specific involving EB formation and then plating of intact EBs on gelatin for 19 days in the presence of 15% FCS with no added cytokines or morphogens. Interestingly, there was no overt defect in the ability of intact MOSPD1‐null EBs to form cardiomyocytes which also involved a non‐specific differentiation assay without the addition of cytokines (Fig. 4E). MOSPD1‐null ESCs differentiated into spontaneously beating cells at comparable frequency to wild type cells with approximately 85% of EBs forming beating cardiomyocytes from both ESC lines (Fig. 4E). Furthermore, deficiency in *Mospd1* expression did not affect the kinetics of cardiomyocyte differentiation—beating regions appeared in the majority of both control EBs and MOSPD1‐null EBs by 10 days (data not shown). To explore the cellular mechanism whereby *Mospd1* functions we assessed the effects of cell density of the level of expression of *Mospd1*. Control E14‐derived MSC‐ like cells grown at high density expressed significantly higher levels of the *Mospd1* implying that cell‐cell contact plays a role in the control of *Mospd1* expression (Fig. 4F).

### Exogenous Expression of MOSPD1, but not MOSPD3, Rescues Differentiation Deficiencies of MOSPD1‐Null ESCs

**Figure 5:**
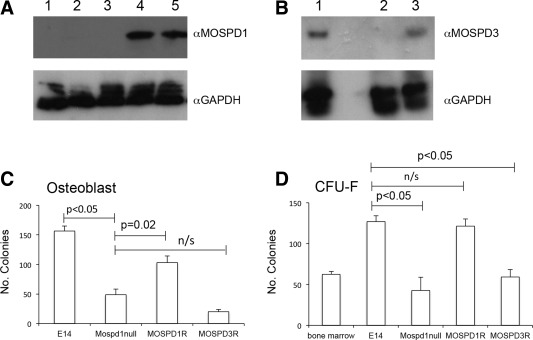
MOSPD1‐null phenotype is rescued by exogenous expression of MOSPD1, but not MOSPD3. **(A):** Western blot of cell lysates from control E14 embryonic stem cells (ESCs) (lane1), *MOSPD1‐null* ESCs (lane 2), *MOSPD1‐null* ESCs transfected with the control CAG empty vector (lane 3) and two *MOSPD1‐null* ESC clones that had been selected after transfection with the CAG‐*Mospd1* vector (lanes 4, 5). Blot was probed with an α‐MOSPD1 antibody and α‐GAPDH antibody as a loading control. **(B):** Western blot analysis of cell lysates from COS cells transfected with the CAG‐*Mospd3* vector (lane1), untransfected E14 ESCs (lane 2) and one of the *MOSPD1‐null* ESC clones that had been selected after transfection with the CAG‐*Mospd3* vector (lane 3). Blot was probed with an α‐MOSPD3 antibody and the α‐GAPDH antibody as a loading control. (C)**:**The number of Alizarin Red‐stained osteoblast colonies generated at day 12 from control E14, MOSPD1‐null and MOSPD1‐null ESCs expressing exogenous MOSPD1 (MOSPD1R) or MOSPD3 (MOSPD3R). **(D):** The number of colony forming unit‐fibrobast colonies generated from control E14, MOSPD1‐null and MOSPD1‐null ESCs expressing exogenous MOSPD1 (MOSPD1R) or MOSPD3 (MOSPD3R). Abbreviation: CFU‐F, colony forming unit‐fibrobast.

To confirm that the deficiencies observed in MOSPD1‐null ESCs were indeed due to loss of the *Mospd1* gene, MOSPD1‐null ESCs were transfected with either a CAG‐*Mospd*1‐IRES‐*Puro* or CAG‐*Mospd*3‐IRES‐*Puro* plasmid. Expression of the MOSPD1 and MOSPD3 proteins, in puromycin‐resistant clones, were confirmed by Western blotting (Fig. 5A, 5B). We used the most robust CFU‐F and osteoblast differentiation assays in our rescue experiments. The number of fibroblast and osteoblast colonies at day 12 in MOSPD1‐null cells transfected with the *Mospd1*‐expressing vector, was significantly higher than the number generated from parental MOSPD1‐null cells (*p* < 0.05) with numbers being restored to levels observed in control E14 ESCs (Fig. 5C, 5D). In contrast MOSPD1‐null cells expressing exogenous Mospd3 protein produced a comparable number of fibroblast and osteoblast colonies to the MOSPD1‐null cells. Thus, expression of exogenous MOSPD1 but not MOSPD3 protein rescued the deficiency observed in MOSPD1‐null cells. These data confirm that MOSPD1 plays an important role in the differentiation of these cell types and refutes the hypothesis that the *Mospd1* and *Mospd3* genes are functionally redundant

### Expression of *Mospd1* in Perivascular MSC Ancestors

Given the apparent role of MOSPD1 in ESC‐derived MSC proliferation we analyzed the expression of this gene in MSC native ancestors isolated from human adipose tissue. Natural forerunners of culture‐defined MSCs have been identified as pericytes 19 and ASC 20. We assessed the expression of a number of genes including *Mospd1* by RNA sequencing in freshly sorted pericytes (CD146+ CD45− CD31− CD34−) and ASCs (CD34+CD146−CD45−CD31−) and then also in these cells following culture for three passages. ASC and pericytes were further subdivided based on Aldefluor staining intensity (ALDH‐dim and ALDH‐bright). Aldeflour is convert to a highly fluorescent product by isoforms of the enzyme ALDH whose presence serves as a presumptive stem cell marker in certain tissue specific and cancer stem cells. Cells staining positive, or bright, for the presence of ALDH were gated in comparison with a negative control population treated with DEAB, an inhibitor of the ALDH enzyme (Supporting Information Fig. S4).

RNA sequencing data confirmed the identify of freshly sorted cells; pericytes expressed high levels of *CD146* compared with ASCs; ASCs expressed higher levels of *CD34* compared with pericytes and ALDH bright ASCs expressed higher levels of ALDH1A1 compared with ALDH‐dim cells (Table 1). There was a higher level of expression of *Mospd1* in the freshly sorted ALDH‐bright compared with the ALDH‐dim supporting the hypothesis that *Mospd1* is expressed in a more stem‐like population. The level of expression of *Mospd1* was significantly increased in the proliferating MSCs that arise from both pericytes and ASCs. In summary, the level of *Mospd1* mRNA expression correlated with the proliferative status of the cells with virtually no expression in freshly sorted pericytes, a slightly higher level in ASCs and a much higher level of expression in MSCs that were cultured from perivascular cells and ASC (Table 1).

**Table 1:** RNA sequencing analysis (expressed as RPMK normalized fragment count numbers) to compare the expression level of *Mospd1* in populations of freshly sorted and cultured ASCs and pericytes

Cell populations	*CD34* (ASC)	*CD146* (PERI)	*RGS5* (PERI)	*CD45*	*ALDH1A1*	*MOSPD1*
**Fresh sorted cells**						
ASC unsorted	2,820	1	1	1	1,051	21
ASC ALDH bright	1,763	1	37	43	1,461	11
ASC ALDH dim	2,388	1	14	45	648	1
Pericytes ALDH dim	23	698	6,526	45	168	1
**Cultured cells**						
ASC ALDH bright	18	16	1	2	1	54
ASC ALDH dim	1	1	1	1	1	51
Pericytes ALDH dim	1	351	35	1	6	69

High levels of expression levels of *CD34* and *CD146/RGS5* confirmed the phenotype of ASCs and pericytes respectively*. CD146, RGS5 and ALDH1A1* confirmed the phenotype of sorted cell populations. (1 = no detectable expression).

Abbreviations: ALDH, aldehyde dehydrogenase; ASC, advential cell; PERI, pericytes.

## Discussion

Our study adds to the growing knowledge about MSP‐domain‐containing proteins and has identified a role for MOSPD1 in the differentiation and proliferation of mesenchymal stem cells derived from ESC in vitro.

A previous study suggested that MOSPD1 plays a pivotal role in the switch between epithelial and mesenchymal cells 12. siRNA knockdown of *Mospd1* mRNA in an osteoblast cell line resulted in increased expression of the epithelial cadherin *Cdh1,* and down‐regulation of its inhibitors, *Snail1* and *Snai2*, and the mesenchymal cadherin *Cdh11* 12. As *Snai1* and *Snai2* are essential for mesenchyme formation and for EMT 21, 22, it was suggested that *Mospd1* was a new player in this process 12. However, our studies using MOSPD1‐null ESCs do not support the hypothesis that MOSPD1 controls the process of EMT per se. We noted that expression of the EMT marker genes *Snai1, Snai2*, and *Cdh11* increased during ESC differentiation in a comparable manner in both MOSPD1‐null and control ESCs indicating that *Mospd1* does not play a role in their regulation. The differentiation phenotype of MOSPD1‐null ESCs suggests that MOSPD1 is involved in the subsequent differentiation and proliferation of MSCs. We noted that MOSPD1‐null ESCs generated significantly fewer colonies than control ESCs when plated in conditions that favored the production of MSC‐like cells (CFU‐F assay) and the production of osteoblasts and adipocytes was also impaired.

MSCs are by definition culture‐selected cells of which native counterparts in the bone marrow and other organs have long remained unknown. Having identified perivascular pericytes and adventitial cells as the origin of the elusive MSCs 23, we wished to determine whether these native MSC ancestors already express *Mospd1*. *Mospd1* mRNA was virtually undetectable in pericytes and adventitial cells purified from human adipose tissue but expression was dramatically augmented in the progeny of these cells after culture, that is, in their MSC descendants. An exception to this however was that freshly sorted ALDH‐bright adventitial cells already expressed *Mospd‐1* at significant levels which could suggest that some of these cells are in a proliferative state in situ. These results further support the hypothesis that MOSPD1 is involved in MSC proliferation. Although the emergence in vivo of bona fide MSCs from pericytes has not been documented, it is accepted that perivascular cells migrate away from blood vessels to participate in tissue renewal and post‐injury repair 24, 25, 26, 27, 28. It will be important to determine whether regenerative perivascular cell mobilization and multiplication at sites of tissue injury is also accompanied by *Mospd1* expression.

We also observed a reduction in the number of hematopoietic progenitors that are generated within EBs derived from differentiating ESCs and speculate that this could be related to the deficiency in differentiation of mesenchymal cells that are known to play a supportive role for hematopoietic progenitors 20, 29. Our findings that the expression of *Mospd1* is higher in cells that are plated at a high density implies that‐cell–cell contact could play a role in the regulation of its expression.

The causal effect of MOSPD1 deficiency was confirmed by rescuing the differentiation defect by exogenous expression of MOSPD1. We previously hypothesized a mechanism of functional redundancy between the *Mospd1* and *Mospd3* genes that might explain the loss of phenotype observed upon continued backcrossing of animals carrying a gene trap integration in the *Mospd3* locus 11. This study apparently refutes this hypothesis as exogenous expression of MOSPD3 is not able to rescue the differentiation deficiencies displayed by MOSPD1‐null cells. However, there could be alternative explanations for the failure of MOSPD3 to rescue MOSPD1 deficiency; for example, if the function of MOSPD3 is dependent on heterodimerization with MOSPD1.

## Conclusion

In conclusion, the generation of *MOSPD1‐null* ESCs lines provides a new tool to study the function of this poorly understood gene family. In contrast to a previous report we show that MOSPD1 is not essential for the transition of epithelial cells to mesenchyme (EMT) but rather it is essential for the subsequent proliferation and differentiation of mesenchymal cells. These findings are supported by the expression profile of *Mospd1* in subpopulations of MSCs and their precursors isolated from human tissue.

## Author Contributions

M.K.: concept and design, collection and assembly of data, data analysis and interpretation, manuscript writing, final approval of manuscript; R.A.A. and S.G.: collection and assembly of data, data analysis and interpretation, final approval of manuscript; M.J.: collection and assembly of data, data analysis and interpretation, manuscript writing, final approval of manuscript; K.B.: concept and design, collection and assembly of data: data analysis and interpretation; A.W.: concept and design, final approval of manuscript; H.T. and B.O.: collection and assembly of data, final approval of manuscript; W.R.H.: collection and assembly of data, data analysis and interpretation, final approval of manuscript; B.P.: concept and design, provision of study material, data analysis and interpretation, final approval of manuscript; L.M.F.: concept and design, data analysis and interpretation, manuscript writing, final approval of manuscript.

## Disclosure of Potential Conflicts of Interest

The authors indicate no potential conflicts of interest.

## Supporting information

Additional Supporting Information may be found in the online version of this article

Supporting Information

Supporting Information

Supporting Information

Supporting Information

Supporting Information

Supporting Information
