# Supplementary figures and images for: A Role for MOSPD1 in Mesenchymal Stem Cell Proliferation and Differentiation
Source: Stem Cells. 2015 Aug 14;33(10):3077–86. doi: 10.1002/stem.2102 (PMC4737116; doi:10.1002/stem.2102)

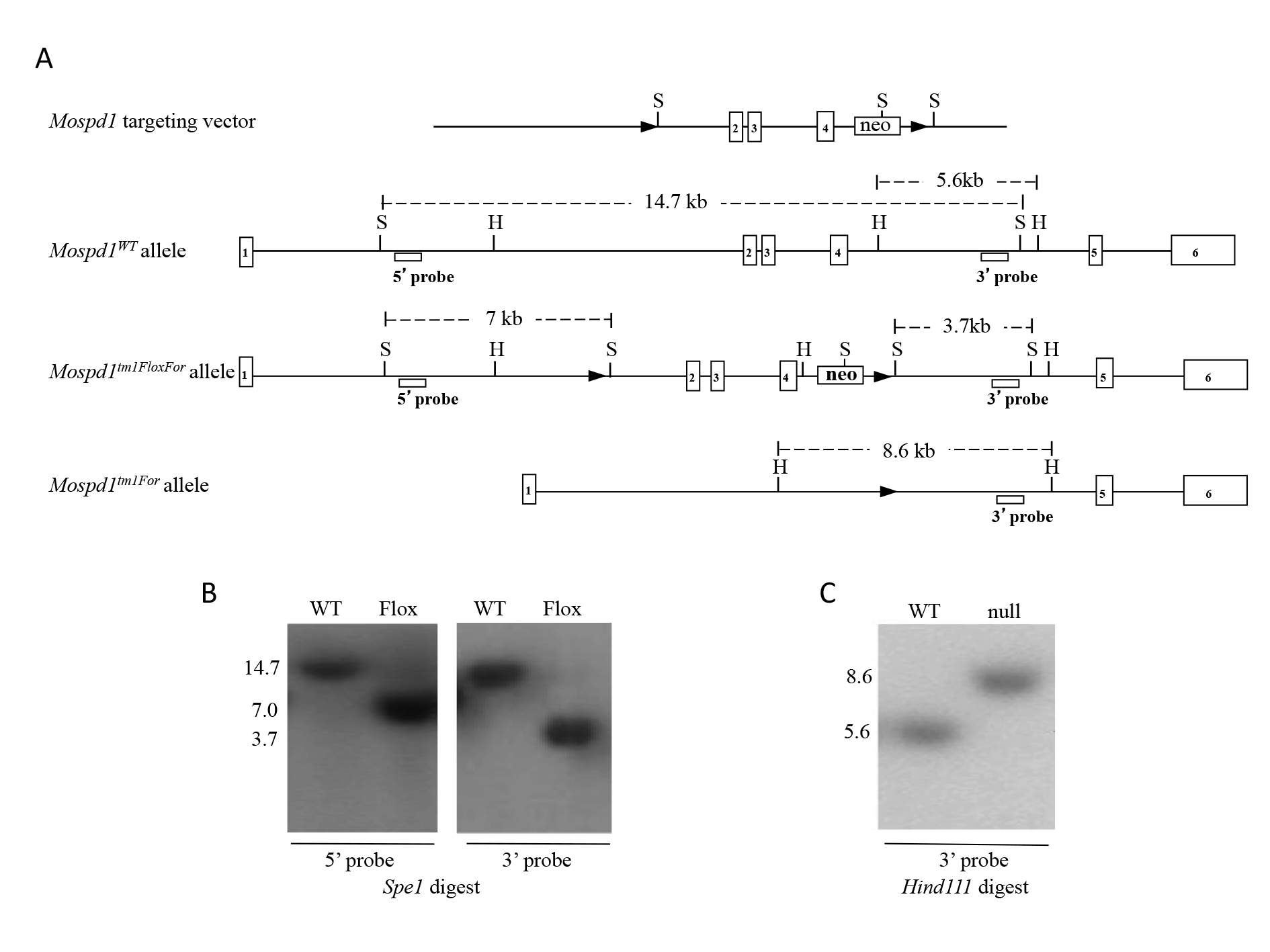

Supplement: Supplementary file 1 — Supporting Information [file STEM-33-3077-s001.tif]

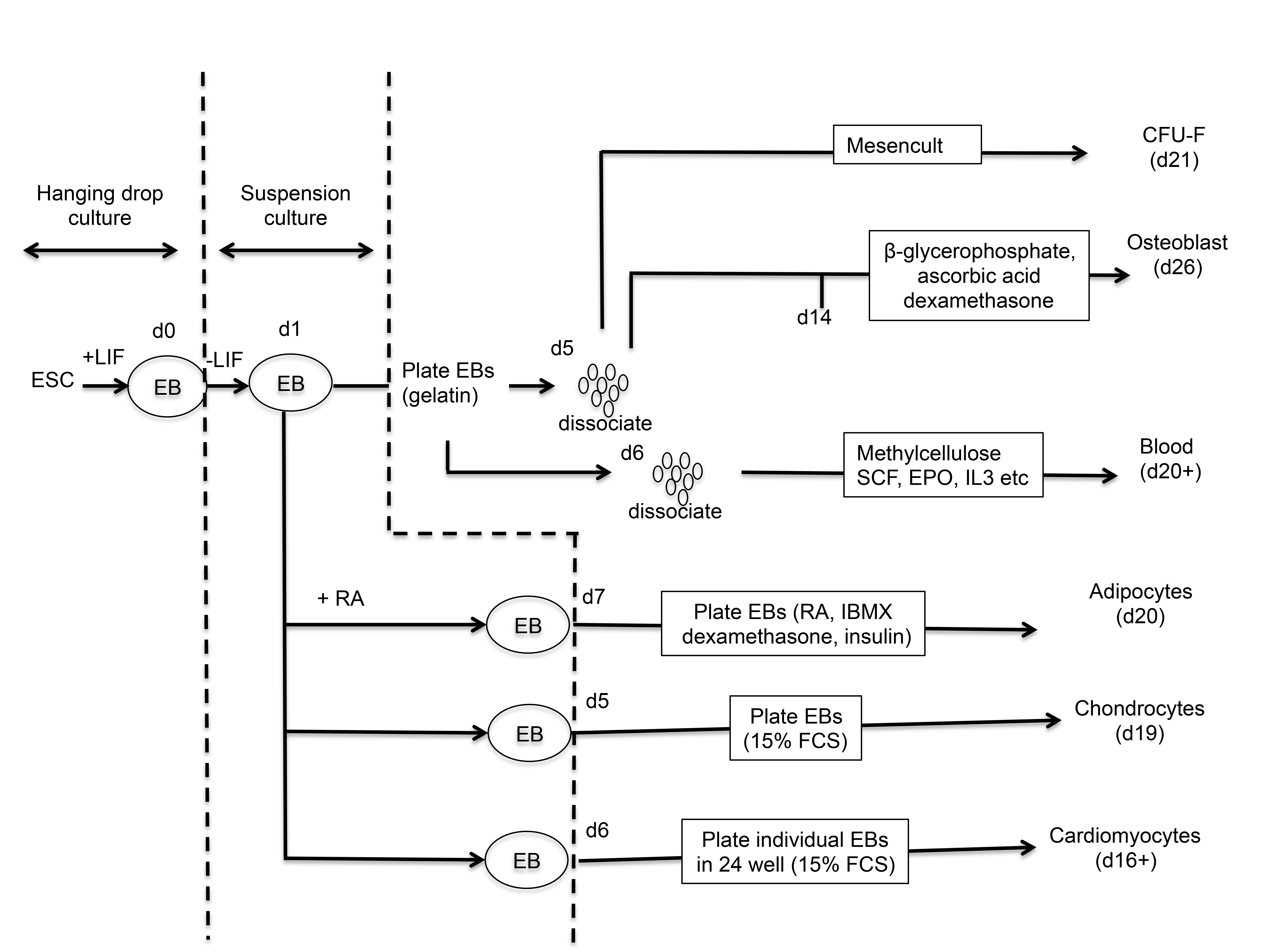

Supplement: Supplementary file 2 — Supporting Information [file STEM-33-3077-s002.tif]

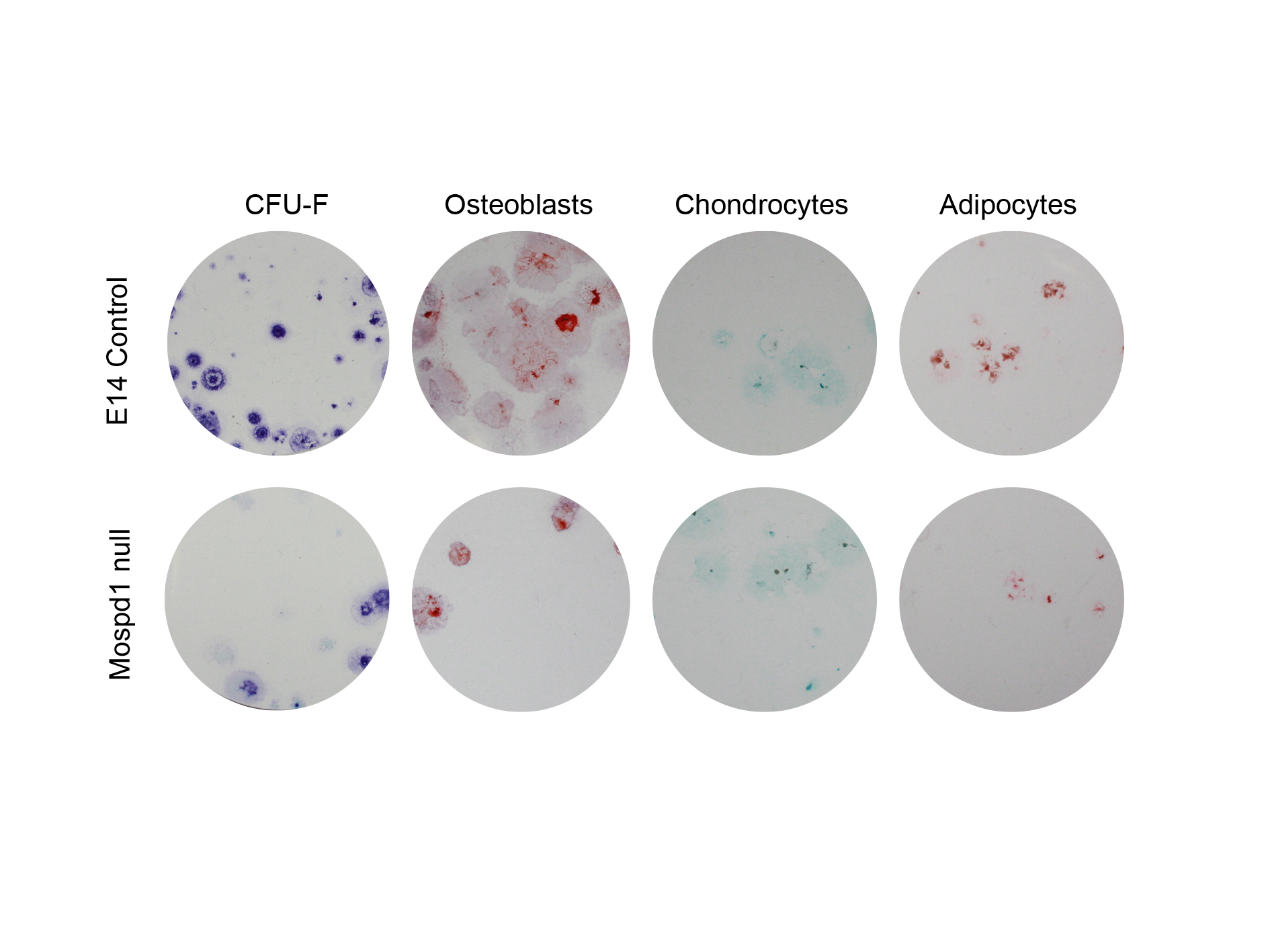

Supplement: Supplementary file 3 — Supporting Information [file STEM-33-3077-s003.tif]

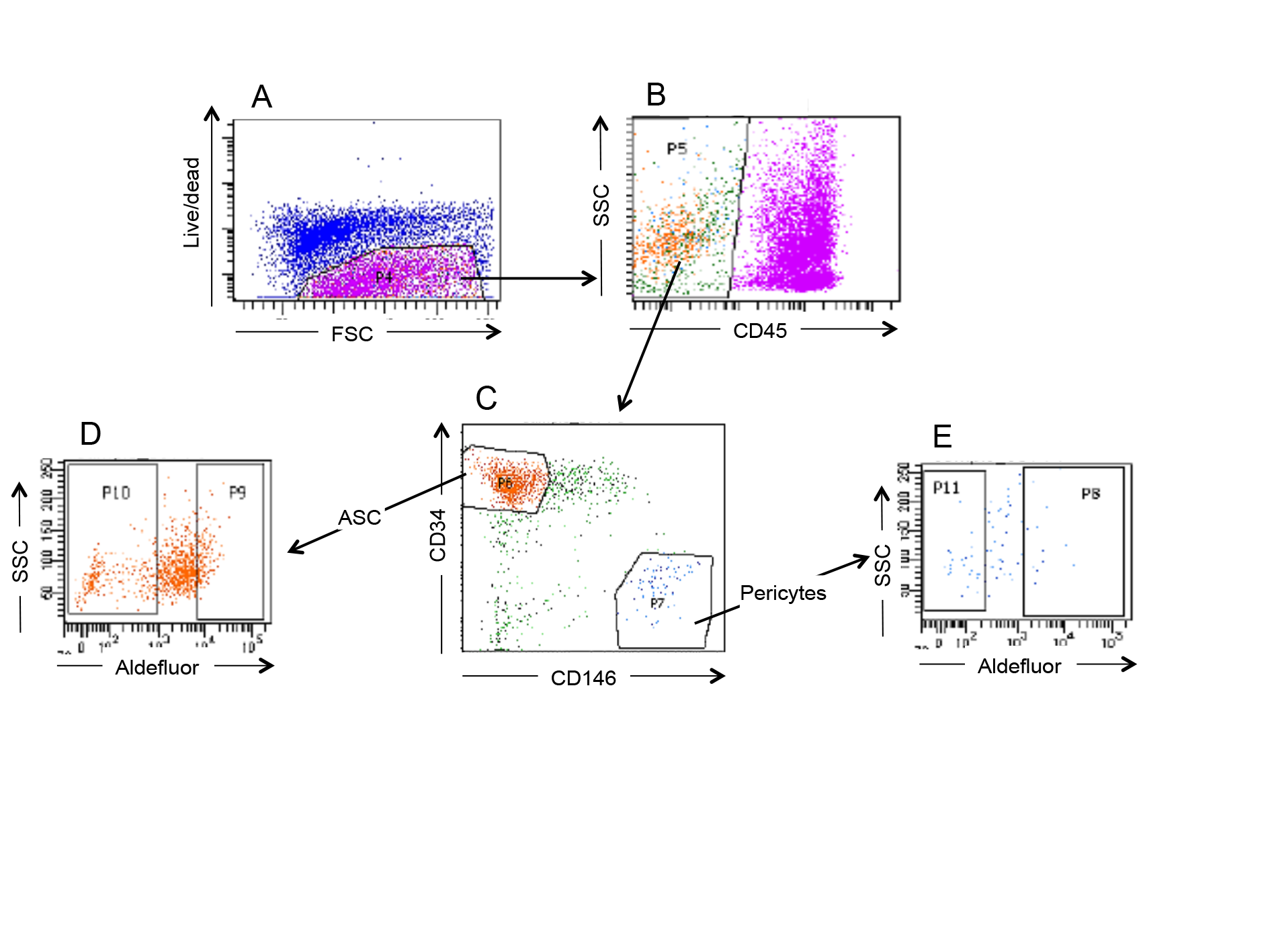

Supplement: Supplementary file 4 — Supporting Information [file STEM-33-3077-s004.tif]
