# Supplementary material for: A Role for MOSPD1 in Mesenchymal Stem Cell Proliferation and Differentiation
Source: Stem Cells. 2015 Aug 14;33(10):3077–86. doi: 10.1002/stem.2102 (PMC4737116; doi:10.1002/stem.2102)
Supplement: Supplementary file 5 — Supporting Information [file STEM-33-3077-s005.doc]

**Supplementary Figures Legends**

**Supplementary Figure S1.  *Mospd1* gene targeting strategy.** Schematic representation of the *Mospd1* targeting vector, the wild type *Mospd1* allele *(Mospd1^WT^),* the *Mospd1* targeted conditional (*Mospd1^tm1FloxFor^*) and the Mospd1-null allele (*Mospd1^tm1For^*) allele after CRE-mediated recombination. A 14.7 kb *Spe1* restriction fragment identified the wild type allele and a 7 kb or 3.7 kb restriction fragment identified the targeted allele using the 5’ or 3’ probe respectively (B). Using the same 3’ probe, a 5.6kb *HindIII* restriction fragment identified the *Mospd1^tm1FloxFor^* allele prior to Cre recombination and a 8.6kb fragment identified the *Mospd1^tm1For^* allele after Cre-mediated excision (C).

**Supplementary Figure S2.** Schematic representation of the differentiation protocol showing the production of day 0 EBs by hanging drops (HD), the subsequent culture of EBs in suspension for 1 day then the dissociation and plating in the presence of various differentiation factors.

**Supplementary Figure S3.** Representative photographs of colonies generated from E14 control or Mospd1-null ESCs after differentiation in CFU-F, osteoblast, chondrocyte or adipocyte differentiation assay.

**Supplementary Figure S4.** Flow cytometry plot generated by FACSDiva (vs 6.1.3) depicting the sorting of lipoaspiration specimens according to immunophenotype and Aldefluor staining intensity. Single cells were selected by two consecutive gates (FSC-width vs FSC-height & SSC-width vs SSC-height; not shown) and then gated for viability (A) and the absence of staining with CD45 (B), a hematopoietic marker. CD45- cells were displayed with respect to CD34 and CD146 expression (C) then ASC (CD34+/CD146-) (D) and pericytes (CD34-/CD146+) (E) that stained dimly (ALDH-dim) and brightly (ALDH-bright) with Aldefluor were collected in 96 well plates for single cell analysis.
