# Supplementary material for: A Role for MOSPD1 in Mesenchymal Stem Cell Proliferation and Differentiation
Source: Stem Cells. 2015 Aug 14;33(10):3077–86. doi: 10.1002/stem.2102 (PMC4737116; doi:10.1002/stem.2102)
Supplement: Supplementary file 6 — Supporting Information [file STEM-33-3077-s006.docx]

**Supplementary Table 1.**

Primer sequences used for the generation of the *Mospd1* targeting vector by recombineering (A), RT-PCR (B) and Q RT-PCR (C). All RT-PCR primer pairs spanned an intron and q-RT-PCR primers and TaqMan Probe were validated prior to use (slope of curve and efficiency).

A

| **Primer name Sequence** | |
| --- | --- |
| Mospd1-loxpneo-1 Forward | 5’- ATGATCCTGATTCAATTCCCAGCACCCATGGCAGCA  CACAGCTATGTAGAACTATGTAGCTCGACCTGCAGCCAAGCTATCG -3’ |
| Mospd1-loxpneo-2 Reverse | 5’- GCACATGACTTTAATCCAACACTCAGAAGGATTCAG  GTACATCTCTTGTGAGTTCCATGACGGCCGCTCTAGAACTAGTG -3’ |
| Mospd1-FRTneo p1 | 5’- GGAGGGTCTAGACTTTCTGCTTAATGCTAGAACTAT  GGACTAGGCCTCAGCGATAAGCTTGATATCGAATTC-3 |
| Mospd1-FRTneo p2 | 5’-CAAAGTTCTCTCTCACTGAAGAGAAAACGGAGGCCCT GTCAGTGAATATTTCTGCGGCCGCTCTAGAACTAG -3’ |

| Gene | Forward primer | Reverse primer | Probe | Intron  span? | Slope | Efficiency |
| --- | --- | --- | --- | --- | --- | --- |
|  |  |  |  |  |  |  |
| FoxA2 | gcggccagcgagttaaag | ctgggactttggtttcctga | atgctgggagccgtg | Y | - 3.5 | 92% |
| Hprt | gctcgagatgtcatgaaggaga | aaagaacttatagccccccttga | ccatcacattgtggccctctgtgtg | Y | - 3.3 | 100% |
| Brachyury | ABI gene expression assay Mm00436877-m1 | | | Y |  |  |
| Mospd3 | agccagacatcttcaggagaat | gcctgccagcaagaagag | UPL#12 | Y | -3.3 | 99% |
| Mospd1 | tgcagtctggagactctgaca | ccaaagcattttggcaatct | UPL #32 | Y | -3.3 | 98% |
| Snail1 | cttgtgtctgcacgacctgt | aggagaatggcttctcacca | UPL #71 | Y | -3.3 | 98% |
| Snai2 | cattgccttgtgtctgcaag | cagtgagggcaagagaaagg | UPL #71 | Y | -3.3 | 98% |
| Pax 6 | gttccctgtcctgtggactc | accgcccttggttaaagtct | UPL #78 | Y | - 4.4 | 69% |
| Cadherin11 | actgcctggctcaacatctc | ctgggactttggtttcctga | UPL #84 | Y | - 3.1 | 107% |

B

| **Primer** | **Forward** | **Reverse** |
| --- | --- | --- |
| *Mospd 1* | GACAACCAGAGTTAGTGGAAGGG | GACAGTTAGCAAACTAGGTCCTG |
| *Mospd 3* | TCAAGGAAAGGGCACAGGGAC | GCACAGAACTCGGAAGCGAAG |
| *β-tubulin* | GGAACATAGCCGTAAACTGC | TCACTGTGCCTGAACTTACC |
| *Nanog* | CAGATAGGCTGATTTGGTTGGTGT | CATCTTCTGCTTCCTGGCAA |
| *Oct4* | GGCGTTCTCTTTGGAAAGGTGTTC | CTCGAACCACATCCTTCTCT |

C
